# Stronger findings from mass spectral data through multi-peak modeling

**DOI:** 10.1186/1471-2105-15-208

**Published:** 2014-06-19

**Authors:** Tommi Suvitaival, Simon Rogers, Samuel Kaski

**Affiliations:** 1 Helsinki Institute for Information Technology HIIT, Department of Information and Computer Science, Aalto University, 00076 Espoo, Finland; 2 School of Computing Science, University of Glasgow, G12 8QQ, Glasgow, UK; 3 Helsinki Institute for Information Technology HIIT, Department of Computer Science, University of Helsinki, Helsinki, Finland

**Keywords:** ANOVA-type modeling, Bayesian modeling, Clustering, Mass spectrometry, Metabolomics, Lipidomics, Nonparametric Bayes

## Abstract

**Background:**

Mass spectrometry-based metabolomic analysis depends upon the identification of spectral peaks by their mass and retention time. Statistical analysis that follows the identification currently relies on one main peak of each compound. However, a compound present in the sample typically produces several spectral peaks due to its isotopic properties and the ionization process of the mass spectrometer device. In this work, we investigate the extent to which these additional peaks can be used to increase the statistical strength of differential analysis.

**Results:**

We present a Bayesian approach for integrating data of multiple detected peaks that come from one compound. We demonstrate the approach through a simulated experiment and validate it on ultra performance liquid chromatography-mass spectrometry (UPLC-MS) experiments for metabolomics and lipidomics. Peaks that are likely to be associated with one compound can be clustered by the similarity of their chromatographic shape. Changes of concentration between sample groups can be inferred more accurately when multiple peaks are available.

**Conclusions:**

When the sample-size is limited, the proposed multi-peak approach improves the accuracy at inferring covariate effects. An R implementation and data are available at http://research.ics.aalto.fi/mi/software/peakANOVA/.

## Background

The study of changes in the levels of metabolites and lipids has become essential for the comprehensive understanding of human health [1]. Chromatography-coupled mass spectrometry (MS) techniques have become the standard method for characterizing the human metabolome [2] and lipidome [3]. The technique generates a spectrum of peaks describing the sample in the plane defined by the retention time from the chromatograph and the mass-to-charge ratio from the mass spectrometer. Each peak in this plane is either generated by an ion arising from one of the compounds present in the sample, or is an artifact of the measurement without association to any of the compounds. The association between the peaks and compounds is unknown *a priori*. The produced peak data are noisy: First, sample preparation introduces sources of uncertainty that propagate to the analysis [4]. Second, the accuracy of the device is limited [5] and it produces biases. Third, peak identification, annotation and pre-processing steps produce additional layers of uncertainty [6]. The effect of errors at all these levels is exacerbated by the “small *n*, large *p*” problem: experiments cover a very limited number of samples, *n*, while the set of compounds measured, *p*, is potentially large.

**Figure 1:**
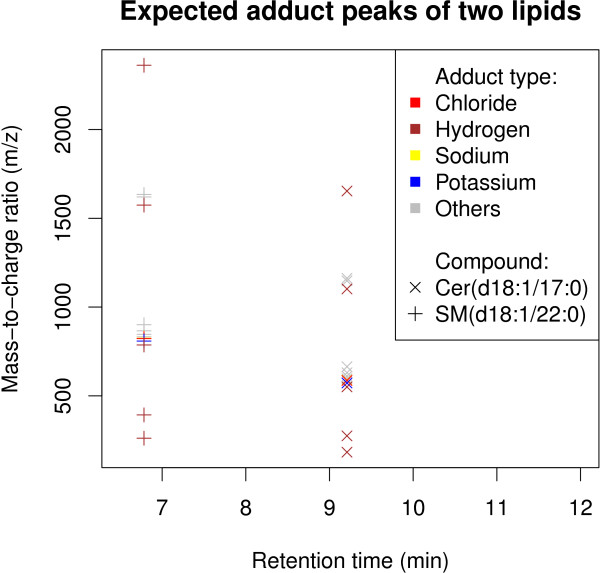
**A schematic of the positions of typical adduct peaks **[7]** in the RT-m/z plane for two lipids, the ceramide Cer(d18:1/17:0) and the sphingomyelin SM(d18:1/22:0).** An adduct peak is formed by an ion attaching to the compound. At the finer detail, each peak in the figure consists of multiple isotope peaks few atomic units apart, as shown for Cer(d18:1/17:0) in Figure 2. Even though the distinct isotope peaks are not visible to the eye here, they are clearly separable by the mass spectrometer. In the figure, adduct types and compounds are marked by colors and characters, respectively.

**Figure 2:**
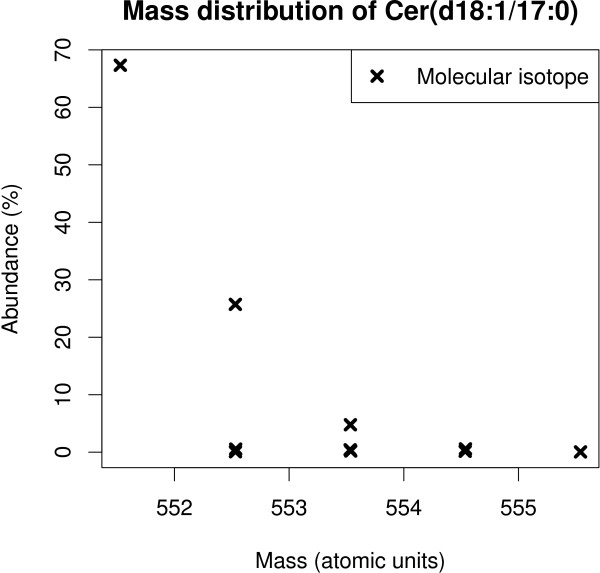
**Natural isotopic distribution of the mass of a typical lipid, the ceramide Cer(d18:1/17:0).** The presence of atomic isotopes leads to the appearance of multiple mass spectral peaks from the compound. Some isotopes are very similar by their mass but still differentiable by the mass spectrometer. The isotope peaks have distinct mass-to-charge ratios at the same retention time (Figure 1).

However, there also is strong informative structure in the data: First, each compound may generate multiple adduct peaks [7] (Figure 1) and isotope peaks [8,9] (Figure 2), whose positions and shapes provide information about the identity of the compound. Second, the concentrations of different compounds generated by or participating in similar biological processes may be highly correlated [10]. An increasing number of machine learning algorithms are being developed for inferring such structure either from raw spectral data [11] or from processed intensity data [12]. The inference of covariate effects—the differences between sample groups determined by the *controlled* covariates of the experiment, such as an intervention—is in the core of the comparative analysis of spectral profiles [13]. In addition to the controlled covariates, confounding factors may affect the observations and are subject to the experiment design. In this work, we focus on inferring effects of the controlled covariates from the data.

**Figure 3:**
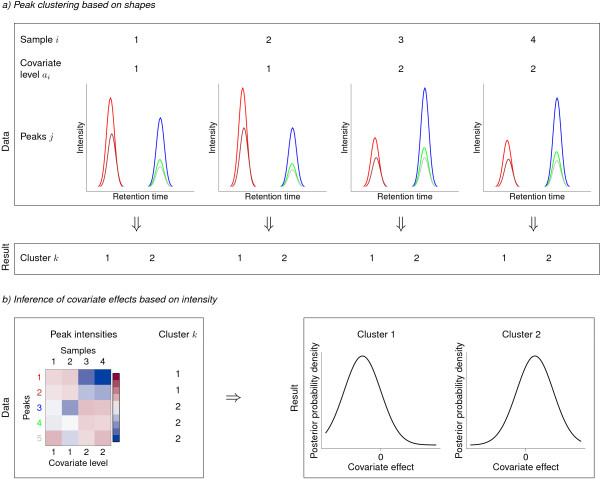
**Flow chart of the method. (a)** Peaks are clustered by their shapes. **(b)** Covariate effects are inferred based on the intensities of the clustered peaks.

The existence of additional peaks in the spectrum is usually seen as a problem and only the main peak of each identified compound is used for further analysis. All peaks are a result of the ionisation process where a charged particle is attached to or detached from a compound. Each such compound-ion pair produces a distinct adduct peak. Random variation in the ionisation process leads to inconsistencies between batches of samples, perceived as variation in the ratio of intensities of the peaks associated with one compound. This is a major source of error for all existing analysis approaches regardless of the choice of the peak used for the analysis. On the other hand, the distribution of the intensities of isotope peaks is by nature well preserved across both samples and compounds. Moreover, the natural isotopic distribution of a compound is known and can be used to make peak annotation more precise. In this way, isotope peaks provide reliable additional information about the differences in compound concentrations between sample groups.We propose a probabilistic approach for extending statistical analysis to all available peaks and demonstrate that the additional peaks can provide a real benefit to the inference of covariate effects (Figure 3). The approach is used to cluster the peaks that are likely to arise from a single compound together and to infer the changes in concentrations of the compounds more accurately based on all these peaks. By this approach, we are addressing the problem of inadequate sample-size by introducing additional data describing the compounds behind the noisy measurements.

To solve the problem we introduce the following assumptions about the generative process of the data within a Bayesian model: First, samples carry between-group differences in their compound concentrations and the differences arise from responses to controlled covariates. Second, multiple observed spectral peaks follow an identical generative process and their heights are a noisy reflection of the true concentration level of the compound. Third, shapes of the peaks from one compound are generated through an identical process following the properties of the measurement device, and thus these shapes are highly similar.

The approach presented in this paper consists of two stages of computational inference: (1) peaks that share a compound as their generative source are clustered together, and (2) the responses to controlled covariates of the experiment are inferred on these clusters of peaks.

The clustering part of the approach is based on a nonparametric Bayesian Dirichlet process model [14]. To improve the performance of this model, we have redefined the prior distributions from a normal distribution to a beta distribution to improve the match to the peak shape similarity observations.

The model for inferring the responses to covariates operates on clusters inferred in the first part. A Bayesian multi-way model [13] is suitable for this task. This model itself could be used for clustering summarized mass spectral intensity data, but in this work, we demonstrate that the clustering can be done more accurately based upon the similarity of chromatographic peak shapes.

## Methods

This section describing the models consists of two parts: clustering of spectral peaks and inference of covariate effects. To maintain the mathematical rigor in the section, we use the terms “samples,” “variables” and “clusters” to refer to the experimental runs of the mass spectrometer, the peaks in the mass spectrometry data, and the yet unknown compounds in the experimental runs, respectively. In the equations, we denote them by the indices

(1) $$\begin{array}{lll} i= & 1,\ldots ,N\;\text{(samples,}\;\mathrm{i.e.},\;\text{experimental runs)},\; & \;\\ j= & 1,\ldots ,P\;\text{(variables,}\;\mathrm{i.e.},\;\text{peaks)},\; & \;\\ k= & 1,\ldots ,K\;\text{(clusters,}\;\mathrm{i.e.},\;\text{compounds)},\; \end{array}$$

respectively, where *N*, *P* and *K* are their respective total numbers. We use bold capital, bold non-capital and non-bold non-capital symbols to refer to matrices, vectors and scalars, respectively (*e.g.*, **V**, **v** and *v*).

### Clusters of peaks based on the similarity

Following earlier work [14], we measure the similarity between the shapes of two peaks by their Pearson correlation computed over a window of retention time after a standard peak alignment [15] across the samples. Truncating negative values to zero, this leads to a distinct similarity matrix **Q**_*i*··_∈[0,1]^*P*×*P*^ for each sample *i*. In the notation, the operator “ ·” indicates that the entire dimension of the array is included, not only the single item *j*. Since a peak is not necessarily observed in every sample, there may be missing values in the matrices. Therefore, we construct an additional mask **R**∈{0,1}^*N*×*P*×*P*^ with binary values $r_{\text{ij}j^{\prime }}$ indicating whether the peak pair (*j*,*j*^′^) in sample *i* appears together within the window where the similarity is measured and whether both of the peaks are observed. An unidentified peak may still be present in the sample below the limit of detection of the mass spectrometer. However, then it is not useful for the inference of covariate effects and, thus, is treated as missing.

#### Model

We assume that the peaks are generated through a Dirichlet process [16]: there is an unknown number of clusters and an unknown and variable number of peaks that arise from each of the clusters. Peaks are assumed to have a one-out-of-many association: each peak is associated with only one of the unknown clusters. With these basic assumptions, we can infer the *P*-by-*K* clustering matrix **V** from the data **Q**. Value *v*_*j* *k*_=1 in the clustering matrix **V** assigns the peak *j* to the cluster *k*. To make the following equations more compact, we use an additional variable, $\varepsilon_{jj^{\prime }}=v_{j\cdot }v_{j^{\prime }\cdot }^{\text{T}}\in \{0,1\}$, which is an inner product of the cluster indicator vectors of the peaks *j* and *j*^′^, to denote whether the two peaks come from the same or different clusters (1 or 0, respectively).

We set a spike-and-slab prior [17] for the peak shape similarity to model the inherent sparse structure of the data. The similarity between any pair of observed peaks (*j*,*j*^′^) is assumed to follow a beta distribution, but the shape of the distribution is assumed to depend on whether the pair comes from the same cluster or from different clusters (shape parameters (*a*_in_,*b*_in_) or (*a*_out_,*b*_out_), when $\varepsilon_{jj^{\prime }}=1$ or 0, respectively). Also the probability of a missing similarity value is assumed to depend on the cluster assignment of the pair ( $p_{0}^{\text{in}}$ or $p_{0}^{\text{out}}$, when $\varepsilon_{jj^{\prime }}=1$ or 0, respectively). The distributional assumptions are

(2) $$\begin{array}{c} q_{\text{ij}j^{\prime }}|\varepsilon_{jj^{\prime }}\;∼\;\;\left\{\;\;\begin{array}{cc} r_{\text{ij}j^{\prime }}\;\left(\;1\;\;-\;p_{0}^{\text{in}}\right)\;\text{Beta}\left(q_{\text{ij}j^{\prime }}|a_{\text{in}},b_{\text{in}}\right)\;+p_{0}^{\text{in}}\delta \left(\;r_{\text{ij}j^{\prime }}\;\right)\;, & \;\varepsilon_{jj^{\prime }}\;=\;1,\\ r_{\text{ij}j^{\prime }}\;\left(\;1\;-\;p_{0}^{\text{out}}\right)\;\text{Beta}\left(q_{\text{ij}j^{\prime }}|a_{\text{out}},b_{\text{out}}\right)\;+p_{0}^{\text{out}}\delta \left(\;r_{\text{ij}j^{\prime }}\;\right)\;, & \;\varepsilon_{jj^{\prime }}\;=\;0, \end{array}\right) \end{array}$$

with the first and the second row of the equation stating the distributions of a peak pair from the same cluster and different clusters, respectively. The likelihood of the entire peak shape data,

(3) $$ℒ\left(Q,R|V\right)=\overset{N}{\underset{i=1}{\prod }}\overset{P-1}{\underset{j=1}{\prod }}\overset{P}{\underset{j^{\prime }=j+1}{\prod }}p\left(q_{\text{ij}j^{\prime }},r_{\text{ij}j^{\prime }}|\varepsilon_{jj^{\prime }}\right),$$

becomes a product over all peak pairs and samples following the distributional assumption of Equation 2.

We further assume that the observed peaks are generated from an unknown finite subset of an infinite set of clusters with an equal prior probability,

(4) $$p\left(\varepsilon_{jj^{\prime }}=1\right)=\frac{1}{P-1+\alpha_{\text{DP}}},$$

for any pair of peaks to be generated from the same cluster. These assumptions define the Dirichlet process, controlled by the concentration parameter *α*_DP_, which determines the prior probability mass outside the existing clusters. Following from this prior assumption, the probability of assigning peak *j* to an existing cluster *k*,

(5) $$\begin{array}{l} p\left(v_{\text{jk}}=1|Q,R,V_{-j,\cdot }\right)\propto s_{k}ℒ\left(Q,R|V_{-j,\cdot },v_{\text{jk}}=1\right), \end{array}$$

becomes weighted by the current size of the cluster, $s_{k}=v_{-j,k}^{\text{T}}v_{-j,k}$. In the notation, matrices **V**_·,-*k*_ and **V**_-*j*,·_ correspond to the matrix **V** with the column *k* and the row *j* omitted, respectively. Alternatively, with probability

(6) $$\begin{array}{l} p\left(v_{j,K+1}=1|Q,R,V\right)\propto \alpha_{\text{DP}}ℒ\left(Q,R|V_{-j,\cdot },v_{j,K+1}=1\right), \end{array}$$

the process may create a new cluster with the index *K*+1 and only the peak *j* inside. Then, the likelihood term is weighted by the Dirichlet process concentration parameter *α*_DP_, which can be seen as a pseudo-count for the number of peaks outside the current *K* clusters.

#### Inference

We infer the posterior distribution of the clustering via Gibbs sampling, which results in a set of *S* samples of the clustering **V**^(*s*)^, *s*=1,…,*S*, from the true posterior distribution *p*(**V**|**Q**,**R**). The computational complexity of a Gibbs iteration is $O\left(KP^{2}\right)$. Further analysis can operate on the entire posterior distribution of the clustering through integration, or on a point estimate of the distribution. We follow earlier work [18] and acquire a point estimate of the posterior distribution of the clustering through finding the least-squares clustering (Section 1 in Additional file 1).

### Covariate effects based on peak heights

Having inferred the grouping of similar peaks into clusters that each correspond to a compound, we infer the differences in concentrations between sample groups for each cluster given the peak height data $X\in R^{P\times N}$ and the clustering **V**. Again, some values in the data may be missing.

#### Model

After a peak-specific centering based on the control group, the observed peak heights for each sample *i* are assumed to be normally distributed with a cluster-specific mean $x_{\cdot i}^{\text{lat}}$:

(7) $$x_{\cdot i}|V,x^{\text{lat}},\sigma^{2}∼N\left(Vx_{\cdot i}^{\text{lat}},\Lambda \right),$$

where the diagonal matrix ***Λ*** contains the peak-specific variance parameters $\sigma^{2}\in R_{+}^{P}$. The cluster-specific means are assumed to be normally distributed with a sample group-specific prior ***α***,

(8) $$x_{\cdot i}^{\text{lat}}|\alpha ,a_{i}∼N\left(\alpha_{\cdot a_{i}},I\right),$$

where *a*_*i*_∈{1,…,*L*_*a*_} is an indicator of group membership (covariate level) for sample *i* and **I** is a *K*-by-*K* identity matrix. The corresponding covariate effects are arranged into an *K*-by- *L*_*a*_ matrix ***α*** and the effects are assumed to be independent and normally distributed,

(9) $$\alpha_{\cdot l}∼\left\{\begin{array}{l} \;\delta \left(\alpha_{\cdot l}\right),\;l=1\\ N\left(0,I\right),\;l=2,\ldots ,L_{a}, \end{array}\right)$$

except for the first level, *l*=1, which is defined as the baseline level and thus is fixed to zero. The model is not limited to one covariate: the cluster-specific mean $x_{i\cdot }^{\text{lat}}$ can be expressed as a sum of effects of multiple covariates and their interaction effects (Section 1 in Additional file 1). Further, the model is readily extensible for dependent covariate effects [19].

The peak-specific variance parameter,

(10) $$\sigma_{j}^{2}∼\text{Scale-Inv-}\chi^{2}\left(n_{0},\sigma_{0}^{2}\right),$$

follows a scaled inverse- *χ*^2^ distribution with *n*_0_ prior samples and a scale $\sigma_{0}^{2}$.

#### Inference and analysis

We infer the covariate effects via Gibbs sampling. Now the clustering matrix **V** has been learned earlier, and is thus taken as known in the model. Computational complexity of a Gibbs iteration is $O\left(\text{NP}K^{2}\right)$. The clustering and the covariate effects can be inferred overnight on a standard desktop computer for a typical-sized data set. The posterior distributions of the covariate effects ***α*** are descriptive of the differences between the sample groups and, thus, interesting from the analysis point of view. To assess the significance of the difference between a sample group, *c*=*l*>1, and the control group, *c*=1, for a cluster *k*, we can study the posterior probability of the effect *α*_*k* *l*_ being greater or less than zero.

### Comparison methods

We call the method described above Model 1. We compared the performance of the following approaches and refer to them as Models 1, 2 and 3: 

1. the multi-peak approach using both peak shape and height information, as proposed in this work (nonparametric clustering of peaks by their shape similarity, inference of covariate effects on the clusters based on the height of the peaks),

2. the multi-peak approach using peak height information only [13] (clustering of peaks and inference of covariate effects based on the height of the peaks only),

3. the typical single-peak approach (inference of covariate effects by the height of the strongest annotated peak only).

For the studied real data sets, we discovered that peak height information alone is not enough for clustering the peaks into individual compounds due to the high level of noise and strong correlations between compounds. Thus, for real data we compared Model 1 to Model 3 and highlight the benefit gained by using peak shape information.

Model 2 assumes the generative Gaussian latent variable model of the Equations 7–10 for the intensity observations **X** and a uniform multinomial prior for the clustering of the peaks. The clustering is inferred by Gibbs sampling together with the covariate effects.

Model 3 quantifies the difference between the covariate level, *c*=*l*, and the control level, *c*=1, as the difference of their means based on the main peak *j*,

(11) $$\alpha_{j,l}=\frac{1}{\overset{N}{\underset{i=1}{\sum }}\delta_{a_{i},l}}\overset{N}{\underset{i=1}{\sum }}\delta_{a_{i},l}x_{j,i}-\frac{1}{\overset{N}{\underset{i=1}{\sum }}\delta_{a_{i},1}}\overset{N}{\underset{i=1}{\sum }}\delta_{a_{i},1}x_{j,i}.$$

The Kronecker delta function $\delta_{a_{i},l}$ selects the samples that have the covariate level *l* by receiving the value 1, when *a*_*i*_=*l*, and 0, otherwise. When the data are log-transformed, the mean difference corresponds to the fold change computed in many analysis platforms such as MZmine [15] and XCMS [6].

### Experiments

We demonstrate the performance of the proposed method through three experiments: a simulated data experiment, a spike-in benchmark experiment with known changes in concentrations, and a gene silencing experiment with measurements of the lipidome of cancer cells.

#### Evaluation measures

Evaluation of the performance on real data sets is not a trivial task, as there is no ground truth available: neither the identity of the peaks nor the true effect sizes are known. Thus, we also used spike-in data, where the true covariate effects are known, although only a small number of the peaks are annotated.

For the simulated and benchmark experiments, we computed the mean squared error (MSE) between inferred and true covariate effects as an evaluation metric. As a result of the log-transformation of the intensity data, we were quantifying relative changes between sample groups, independent of the average height of each peak. In the model, we thus assumed that the change is preserved across the peaks of one compound, in relative terms. The significance of the difference in the MSE of the proposed approach and the comparison method was tested by the paired one-sided *t*-test. The false discovery rate was controlled by the Benjamini-Hochberg step-up procedure [20]. Additionally for the simulated experiment, we studied the inference of the statistical significance of effects, since the true distribution of the data was known.

To assess the sensitivity of the approaches to noise in natural lipidomic data lacking a ground truth, we used two types of indirect evaluation: First, we studied the consistency of the inferred covariate effects given a prior assumption about their similarity. Second, we examined the robustness of the inferred covariate effects to noise. Finally, we demonstrated differences between the multi-peak and single-peak approaches through examples of qualitative analysis of annotated peak clusters.

#### Simulated data

We started by investigating the performance of the proposed approach on synthetic data, where the true covariate effects are known. We focused on a usual task in exploratory analysis of biological data: the detection of significant non-zero covariate effects. We measured the performance by the accuracy at this task—the ratio of true positive and true negative significant differences among all effects. We used the 95% posterior quantiles to determine the significance. Additionally, we compared the approaches by the MSE to the true effects and studied the performance of the two clustering models by computing the normalized information distance (NID) [21] to the true clustering.

The approaches were tested across a set of potential experimental settings to study how the observation of additional peaks and samples affects the performance. Simulated data were generated by assuming the latent structure of Model 1. The following data parameters were varied on a grid: sample-size *N*=2×{3,7,15} and peak-specific noise *σ*^2^={1,5}. Additionally, the number of peaks per cluster was varied between 3, 7 and 15. Covariate effects ***α***_·2_=[2,-1,0.5,0,0,0,0] were generated for each data set. The experiment was repeated 100 times with independent data sets. The results are reported in the Results and discussion section.

#### Benchmark data with known changes in concentrations

The benchmark data set of apple samples [22] includes a set of annotated spike-in compounds with increases of 20, 40 or 100% in concentrations. We started with the raw spectral data [23] in order to acquire the shapes of the peaks in addition to their heights. The mass spectra were pre-processed using MZmine 2 [15] (Section 4 in Additional file 1). We used standard pre-processing methodology also used in the original publications of the data sets, thus maintaining the focus of the work on the potential benefit gained from the multiple peaks. The compared approaches were on the same line in terms of the data.

We evaluated the approaches by the MSE between inferred and true covariate effects. If the cluster contained multiple annotated peaks, the effect of each annotated peak was evaluated separately for the single-peak approach. Clusters with no annotated peaks were considered to have a 0% true effect and the effect of the single-peak approach was inferred based on the strongest peak of the cluster.

#### Lipidomic data from a gene silencing study

The data come from a recent experiment [24] to study the effects of gene silencing treatments on lipidomic profiles and growth of breast cancer tissue. Driven by the lack of ground truth about the covariate effects, we evaluated the inferred effects indirectly in two ways: (1) by quantifying the consistency of the effects within a lipid family and (2) by quantifying the robustness of the magnitudes of the inferred effects across the lipidome in presence of additional noise. Additionally, we investigated the stability of the inferred clustering on the data and qualitatively analyzed differences between the covariate effects of single peaks and the effects inferred on clusters of peaks by Model 1.

The data included 32 lipidomic profiles of breast cancer cells from the ZR-75-1 cell line. We inferred the effects of seven distinct silencing interventions on metabolism- regulating genes (Section 5 in Additional file 1) at two time points. The raw spectra were pre-processed with MZmine 2 as described in the original publication [24], in addition to which the shape similarities of the peaks were computed.

##### Consistency of effect signs

In the first task, we quantified the consistency as the accuracy at predicting the covariate effect of a test lipid given the model on the covariate effects of other lipids of the same family. For instance, we predicted the effect of a gene silencing treatment on the sphingomyelin SM(d18:1/22:0) based on the sphingomyelin compounds in the training set. We examined the sign of the effect instead of the absolute effect, since even within a family of lipids the changes have a high variance and thus cannot be reliably predicted without imposing additional information about the biological system.

We predicted the signs of the covariate effects for test lipids in a three-fold cross-validation setting with 100 randomizations. The examined lipids included the annotated members from the three most abundant families of lipids that had two or more peaks identified with the clustering model (Section 5 in Additional file 1).

Further, we studied the influence of noise to the consistency by adding independent normally distributed noise (from *σ*=0 to *σ*=10) on the peak intensity observations. Added noise variance *σ*=1 was equal to the existing original variance in the data, and the upper bound for the signal-to-noise ratio then was 0.5 (Additional file 1: Table S4).

##### Robustness of effect magnitudes

To evaluate the inferred effects at the scale of the entire observed lipidome, we examined the consistency of inferred covariate effects between the original and noise-added data sets. This experiment simulated the situation where the true effects are known (effects from the original data set), but the data based on which the effects are inferred are noisy (the added-noise data set). To measure the consistency, we computed the Spearman correlation between the covariate effects inferred from the original and the added-noise data sets. We studied all clusters with two or more peaks, constituting 20% of the clusters.

## Results and discussion

### Simulated data

**Figure 4:**
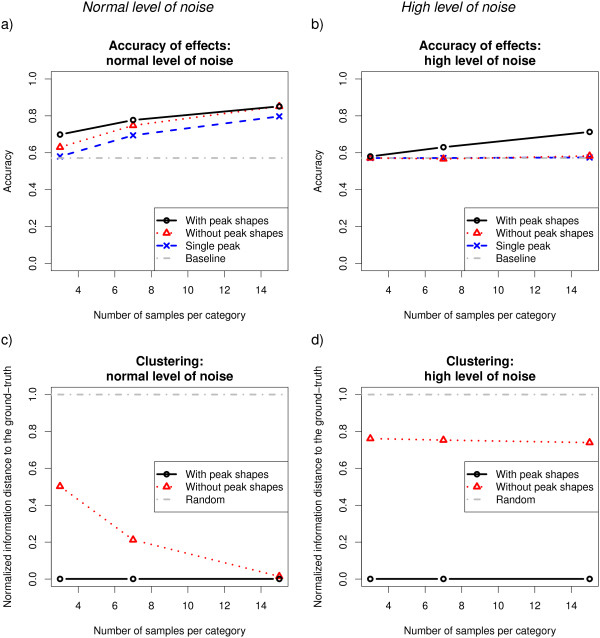
**The use of data from multiple peaks and the peak shape information increased the accuracy at detecting significant covariate effects on simulated data.** Accuracy of Models 1, 2 and 3 for simulated data is shown as a function of the sample-size in two settings: normal and high level of noise (left: *σ*^2^=1, and right: *σ*^2^=5, respectively). Top **(a-b)**: Accuracy at inferring the significance of the generated covariate effects. Bottom **(c-d)**: Normalized information distance (NID) between the inferred and the true clustering. An entirely random and an exactly correct clustering correspond to a NID of 1 and 0, respectively.

On a normal level of noise (*σ*^2^=1), the multi-peak approaches (Models 1 and 2) always performed better at detecting significant covariate effects than the single-peak approach (Model 3; Figure 4a) and only with enough samples the performance of Model 2 became comparable to Model 1. The inferred clustering of Model 1 was perfect while the clustering performance of Model 2 heavily depended on the number of samples available (Figure 4c).

On a high level of noise (*σ*^2^=5), only Model 1 worked (Figure 4b). The reason for the failure of Model 2 was the imperfectly inferred clustering (Figure 4d). The good performance of Model 1 resulted from the clustering step, which is robust to noise in the peak heights. The peak shape similarity gave strong evidence for inferring the clusters already from a single sample.

**Figure 5:**
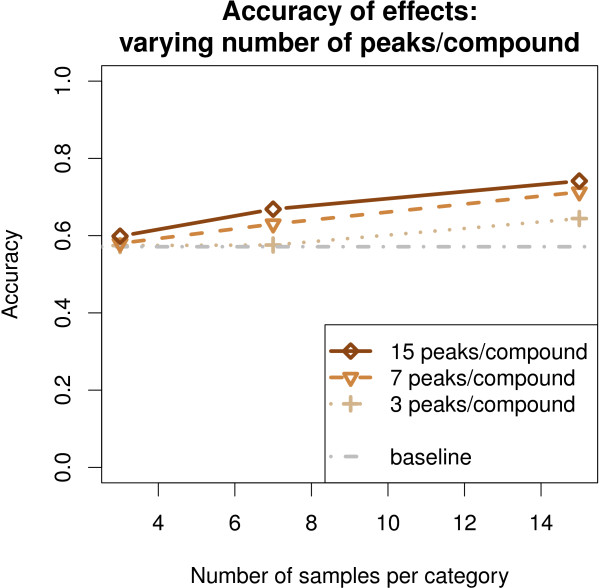
**The performance of Model 1 improved when more peaks per compound were available in the simulated data.** The curves show the accuracy as a function of sample-size for simulated data with 15, 7 and 3 peaks per compound.

The MSE between the inferred and true covariate effects for Model 1 was smaller compared to Model 3 in all the 24 setups of the experimental grid (Additional file 1: Table S1). The difference was statistically significant in 22 setups and in all setups at the high level of noise.The performance of Model 1 clearly improved, when more peaks from a cluster were present in the data (Figure 5). This was pronounced at a high level of noise, when the observation of a single peak is unreliable for inferring the covariate effects. In a similar way as in averaging over samples, the model is able to overcome peak-specific noise also by averaging over multiple peaks.

### Benchmark data with known changes in concentrations

In the first demonstration on real UPLC-MS data [22], we show that Model 1 can infer the artificial perturbations in a spike-in experiment more accurately than the single-peak approach.

In the positive ion mode, the model inferred 794 clusters, among which 135 clusters included more than one peak. Seven clusters included annotated peaks from the spike-in compounds, four of which included more than one annotated peak (Additional file 1: Table S2). Peaks from two compounds were distributed to two and four clusters, respectively. In the negative ion mode, the model inferred 367 clusters, among which 113 clusters were non-singletons. Three clusters included annotated peaks from the spike-in compounds, all of these clusters included more than one annotated peak and all peaks from one compound were clustered together. In both the ion modes, all clusters with annotated peaks were specific to one compound.

**Figure 6:**
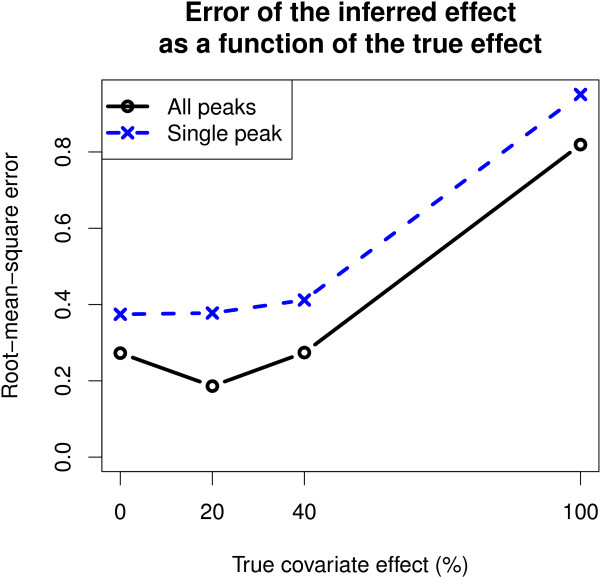
**Model 1 had a more accurate quantification of the covariate effects for the spike-in compounds as well as for the unchanged non-annotated compounds in the benchmark experiment.** Root-mean-square error (RMSE; y-axis) between the inferred and true covariate effects is smaller for Model 1 (All peaks) than for the single-peak approach (Single peak) at all the magnitudes of the true effect (x-axis). Differences were statistically significant for changes of 0 to 40% (Additional file 1: Table S3).

Model 1 had a lower error than Model 3 at all magnitudes of the true effect with the strongest relative improvement occurring at the small magnitudes (Figure 6). The difference was statistically significant for covariate effects from 0 to 40% (Additional file 1: Table S3).

### Lipidomic data from a gene silencing study

In the second demonstration on real UPLC-MS data [24], we show that Model 1 can infer more consistent covariate effects in two ways even though the true effects are unknown.

#### Consistency and robustness of effects

**Figure 7:**
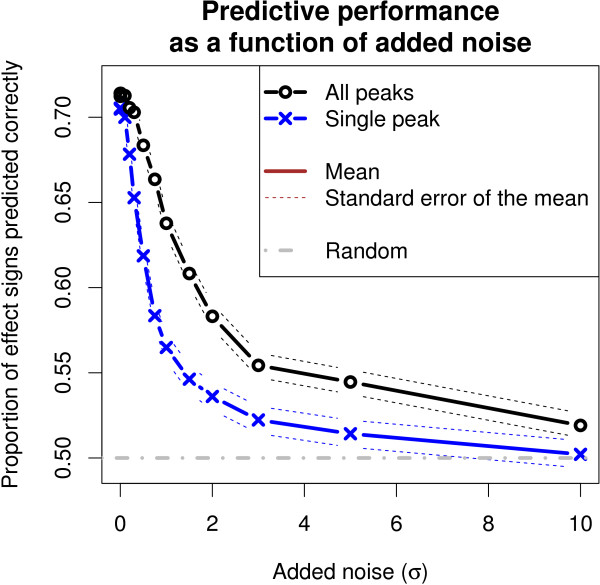
**Model 1 (All peaks) had a better accuracy at the prediction of signs of covariate effects for previously unseen lipids in the lipidomic gene-silencing data set compared to Model 3 (Single peak).** The difference became pronounced when simulated noise was added to the data. The prediction was based on the inferred covariate effects of compounds from the same lipid family and was done in a cross-validation setting. In the task, the effects of the seven gene-silencing treatments were predicted on the three most abundant families of lipids in two time points. Points *σ*=0 and *σ*>0 on the x-axis show the prediction accuracy (y-axis) for the original data and the data with added noise, respectively.

**Figure 8:**
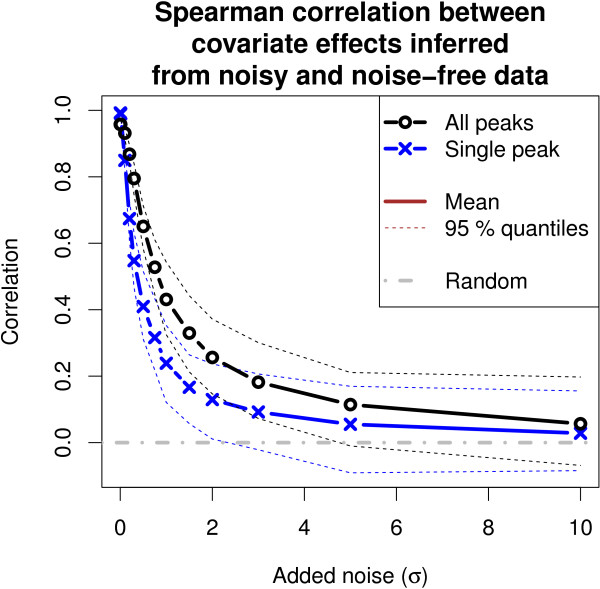
**The covariate effects inferred by Model 1 (All peaks) were more robust to noise compared to Model 3 (Single peak).** At moderate levels of noise, which is the regime of many biological experiments, the confidence intervals over 100 randomizations did not overlap at all. The robustness was quantified as the Spearman correlation (y-axis) between the effects inferred from the noisy and noise-free versions of the lipidomic gene silencing data set as a function of the level of noise (x-axis).

When examining the consistency of effects within a lipid family, Model 1 was more consistent than Model 3 at all levels of noise (Figure 7). When no noise was added and also at moderate levels of noise, both approaches performed clearly better than expected by random chance. When noise was added, Model 3 suffered more and its performance reduced to the random level more rapidly. Given the assumption about the general similarity of lipids within a family is true, Model 1 inferred the covariate effects more consistently.When examining the robustness of effect magnitudes, Model 1 was more consistent than Model 3 when noise was added to the data (Figure 8). The confidence intervals from the 100 randomizations did not overlap at all at moderate levels of noise.

#### Stability

Since the proposed approach is sensitive to the inferred clustering of the data, we analyzed the stability of the inferred clustering on biological data, using the lipidomic gene silencing data as a case study. We tested the influence of the concentration parameter *α*_DP_ in the Dirichlet process clustering model. The clustering result for the lipidomic gene silencing data was robust to changes in the magnitude of the concentration parameter (Additional file 1: Figure S2). As expected, the number of clusters increased, when the preset value of the concentration parameter increased, but the relative change was small.

#### Qualitative analysis

**Figure 9:**
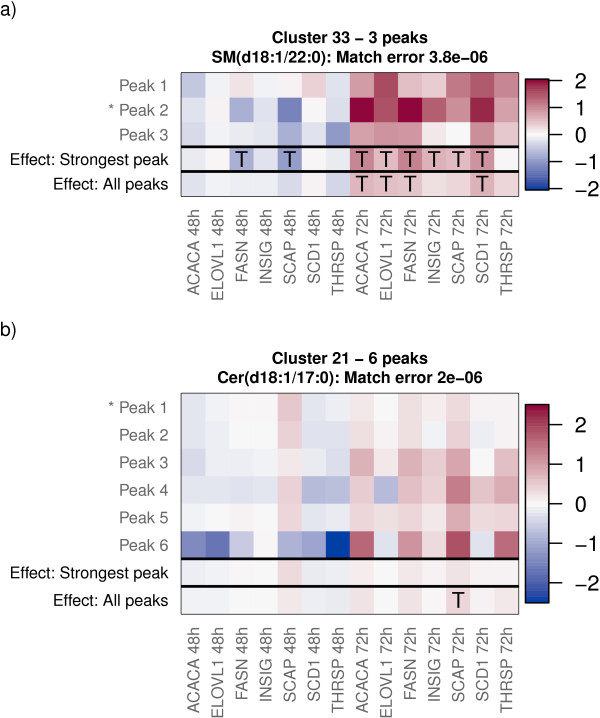
**Example clusters of peaks from the lipidomic gene silencing data with differences in the covariate effects inferred based on a single peak and multiple peaks.** The heat maps show changes in the lipid concentrations driven by the gene silencing interventions (columns). Covariate effects inferred by Models 3 and 1 using a single peak and all peaks, respectively, are shown on the two bottom rows of each heat map. The log2 fold changes of each peak associated with the compound are shown on the top rows. Changes that by the magnitude fall to the top-5% across the entire observed lipidome are highlighted by the symbol ”T.“ Top **(a)**: The sphingomyelin SM(d18:1-22:0) with three peaks. Many strong changes for SM(d18:1-22:0) became weaker when they were inferred based on all three peaks. Bottom **(b)**: The ceramide Cer(d18:1-17:0) with six peaks. The effect of the SCAP silencing for Cer(d18:1-17:0) at 72 hours became strong when it was inferred based on all six peaks.

Finally, we give concrete examples of potential findings that the approaches can uncover and demonstrate how analysis based on a single peak may lead to a different outcome depending on the choice of the peak.The intervention-driven changes of individual peaks from two lipids along with the covariate effects inferred by Models 1 and 3 are shown in Figure 9. In the case of the sphingomyelin SM(d18:1/22:0), there were strong covariate effects inferred by Model 3 but many of these effects became weaker when inferred based on multiple peaks by Model 1. On the contrary, Model 3 inferred weak covariate effects for the ceramide Cer(d18:1/17:0) but based on multiple peaks and Model 1, one of the effects was actually among the top-5% strongest effects across the observed lipidome.

## Conclusions

We have empirically demonstrated that a model-based integration of multiple peaks can lead to an improved accuracy in the inference of covariate effects, and we introduced a novel method for this task. While the sample-size is always restricted by external constraints such as the experiment budget or the availability of suitable patients, the inference based on multiple peaks gives a shortcut to extracting more information from the limited set of samples, thereby directly addressing the “small *n*, large *p*” problem. However, some types of systematic measurement error, such as some batch effects, escape this treatment and can only be reduced by introducing independent replicates. Based on the results presented in this work, we argue that additional peaks are especially useful when the signal-to-noise ratio is low and the differences between sample groups are small.

We suggest that all the detected peaks that can be associated with a compound should be taken into account in the comparative analysis. This is possible through the two-step generative modeling approach presented in this work: (1) by identifying the peaks that can be associated with one compound through clustering the peaks based on their shape similarity and (2) by the inference of covariate effects on the clusters, each representing one compound.

## Abbreviations

ANOVA: Analysis of variance; Cer: Ceramide; SM: Sphingomyelin; UPLC-MS: Ultra performance liquid chromatography-mass spectrometry.

## Competing interests

The authors declare that they have no competing interests.

## Authors’ contributions

The method was developed jointly by TS, SK and SR. TS had a lead role at implementing the model, designing and implementing the experiments, and at preparing the manuscript. All authors read and approved the manuscript.

## Supplementary Material

Additional file 1 **Supplementary material.** More details of the experiments. Click here for file
