# Supplementary material for: Stronger findings from mass spectral data through multi-peak modeling
Source: BMC Bioinformatics. 2014 Jun 19;15:208. doi: 10.1186/1471-2105-15-208 (PMC4080774; doi:10.1186/1471-2105-15-208)
Supplement: Additional file 1 — Supplementary material. More details of the experiments. [file 1471-2105-15-208-S1.pdf]

Supplementary material: Stronger findings from mass spectral data through multi-peak modeling

Tommi Suvitaival*[1], Simon Rogers†[2] and Samuel Kaski‡[1,3]

[1]Helsinki Institute for Information Technology HIIT, Department of Information and Computer Science, Aalto University
[2]School of Computing Science, University of Glasgow
[3]Helsinki Institute for Information Technology HIIT, Department of Computer Science, University of Helsinki

May 29, 2014

Abstract

This document is the Supplementary Material document for the publication titled *Stronger findings from mass spectral data through multi-peak modeling* [6].

An R implementation and data are available at http://research.ics.aalto.fi/mi/software/peakANOVA/.

1 Model

1.1 Clusters of peaks based on the similarity

1.1.1 Dirichlet process prior

- Dirichlet process assumes an infinite set of clusters and that the observed data are generated from an unknown finite subset of these clusters.
- The Dirichlet process assumes an equal prior probability

$$p\left(\varepsilon_{jj'} = 1\right) = \frac{1}{P - 1 + \alpha_{\mathrm{DP}}} \tag{1}$$

  for any pair of peaks, $(j, j') \in 1, \ldots, P$, to be generated from the same cluster. In the equation, α_{DP} is the Dirichlet process concentration parameter.

- The prior probability for assigning a peak into:

*tommi.suvitaival@aalto.fi
†simon.rogers@glasgow.ac.uk
‡samuel.kaski@aalto.fi

– a cluster k with s_k existing members is

$$p\left(v_{j,k}=1|\mathbf{V}_{-j,\cdot}\right)=\frac{s_k}{P-1+\alpha_{\mathrm{DP}}}, \tag{2}$$

giving the weight s_k for the assignment probability;

– a new cluster $K+1$ is

$$p\left(v_{j,K+1}=1\right)=\frac{\alpha_{\mathrm{DP}}}{P-1+\alpha_{\mathrm{DP}}}, \tag{3}$$

where the Dirichlet process concentration parameter α_{DP} can be seen as a pseudo count for peaks outside the current K clusters. It controls the probability at which the process assigns peaks into a previously empty cluster.

- Following from the exchangeability property, the posterior probability is invariant to the order at which the peaks are assigned to the clusters.

1.1.2 Point estimate for the posterior distribution

We follow earlier work [2] and acquire a point estimate of the posterior distribution of the clustering through finding the least-squares clustering (Section 1 in Additional file 1): the posterior sample s whose adjacency matrix $\mathbf{V}^{(s)}\mathbf{V}^{(s)\mathrm{T}}$ has the smallest squared deviation from the posterior pairwise probabilities $\mathbf{\Pi}$ of the peaks. The index of the least-squares clustering thus is

$$s_{\mathrm{LS}}=\underset{s\in\{1,\ldots,S\}}{\arg\min}\sum_{j=1}^{P-1}\sum_{j'=j+1}^{P}\left(\mathbf{v}_{j\cdot}^{(s)}\mathbf{v}_{j'\cdot}^{(s)\mathrm{T}}-\widehat{\pi}_{jj'}\right)^2, \tag{4}$$

where the posterior pairwise probabilities

$$\widehat{\mathbf{\Pi}}=\frac{1}{S}\sum_{s=1}^{S}\mathbf{V}^{(s)}\mathbf{V}^{(s)\mathrm{T}} \tag{5}$$

are estimated as the average adjacency matrix over the posterior samples.

1.2 Covariate effects based on peak heights

1.2.1 Multi-way model

For an arbitrary number of covariates, the cluster-specific means (Equation 8 in the publication)

$$\mathbf{x}_{\cdot i}^{\mathrm{lat}}|\boldsymbol{\alpha},a_i\sim\mathcal{N}\left(\boldsymbol{\alpha}_{\cdot a_i},\mathbf{I}\right), \tag{6}$$

can be expressed as a sum of individual covariate effects and the interaction effects of two or more covariates. This generalization can be written as

$$\mathbf{x}_{\cdot i}^{\mathrm{lat}}|\mathbf{E},\mathbf{C}\sim\mathcal{N}\left(\mathbf{E}\mathbf{c}_{\cdot i},\mathbf{I}\right), \tag{7}$$

where $\mathbf{C}$ is an L-by-N binary matrix, whose column i indicates the known levels of all the covariates and all their resulting interaction levels for sample i.

The matrix $\mathbf{E}$ has dimensions K-by-L and its row k contains the effects $\mathbf{e}_{k\cdot}$ of the respective covariates and their interactions for cluster k. Similarly, Equation 9 in the publication [6],

$$\boldsymbol{\alpha}_{\cdot l} \sim \begin{cases} \delta\left(\boldsymbol{\alpha}_{\cdot l}\right), & l = 1 \\ \mathcal{N}\left(\mathbf{0}, \mathbf{I}\right), & l = 2, \ldots, L_a, \end{cases} \tag{8}$$

can then be generalized as

$$\mathbf{e}_{\cdot l} \sim \delta\left(\mathbf{e}_{\cdot l}\right), \tag{9}$$

when any of the covariates is at the base level, and

$$\mathbf{e}_{\cdot l} \sim \mathcal{N}\left(\mathbf{0}, \mathbf{I}\right) \tag{10}$$

otherwise.

1.2.2 Computational complexities

- Computational complexity expressed in terms of the number of samples, N, the number of peaks P and the number of clusters K.
- Model 1:
  - Pre-computation of the likelihood values based on the peak similarity: $\mathcal{O}\left(NP^2\right)$,
  - Clustering of peaks based on the similarity, a Gibbs sample: $\mathcal{O}\left(KP^2\right)$,
  - Inference of covariate effects based on the peak heights, a Gibbs sample: $\mathcal{O}\left(NPK^2\right)$;
- Model 2:
  - Clustering and inference of covariate effects based on the peak heights, a Gibbs sample: $\mathcal{O}\left(KPN^2 + NPK^2\right)$;
- Model 3:
  - Inference of covariate effects based on the height of a single peak: $\mathcal{O}\left(NP\right)$.

2 Software versions

- MZmine 2.9.1 [5] modified to export peak shape correlations
  - The modified version is available at http://research.ics.aalto.fi/mi/software/peakANOVA/
- R 2.15.1
- PeakANOVA package
  - Available at http://research.ics.aalto.fi/mi/software/peakANOVA/

3 Simulated data

3.1 Data generation

- Data were generated from Model 1 with same parameters as with which the learning was done
- Example illustration of an distribution of the peak shape similarity values: Figure 1

3.2 Inference of the clustering

- Burn-in: 1,000 Gibbs samples
- Sampling after burn-in: 1,000 Gibbs samples

3.3 Inference of covariate effects

- Burn-in: 10,000 Gibbs samples
- Sampling after burn-in: 10,000 Gibbs samples
- Thinning: Every 10th Gibbs sample saved

3.4 Significance of covariate effects

- Inferred covariate effect was deemed significantly positive/negative if at least 95 % of the probability mass of the posterior distribution was above/below zero

3.5 Test of difference between the approaches

- Posterior mean of the change computed for each cluster/peak from the Gibbs samples
- Squared error to the ground-truth change (0, 0.5, -1, or 2) is computed for each cluster/peak by both the approaches
- Statistical significance of the difference between the mean squared errors (MSE) of the two approaches is tested by one-sided paired t-test
  - Null hypothesis: No difference between the MSEs
  - Alternative hypothesis: The MSE of Model 1 is smaller
  - False discovery rate of the test was controlled by the Benjamini-Hochberg step-up procedure [1] at level 0.01
- Result shown in Table 1.

Table 1: Model 1 (All peaks) yields a more accurate quantification of the covariate effects in all the settings of the simulated data when compared to Model 3 (Single peak). The difference in the root mean squared error (RMSE) between the two approaches is significant in 22 out of the 24 comparisons. The one sided paired t-test is controlled for the false discovery with the Benjamini-Hochberg correction at the level 0.01. Significant differences at confidence levels 95 % and 99 % are highlighted by symbols "*" and "**," respectively. The number of samples per category (N) and the noise level (σ^2) in the simulated data are shown in the leftmost column.

**Simulated data setting**	**True covariate effect**	**RMSE Single peak**	**RMSE All peaks**	**Adjusted p-value for the difference**
$N = 3$, $\sigma^2 = 1$				
	0	0.87	**0.65**	$2.3 \times 10^{-7**}$
	0.5	0.98	**0.61**	$7.4 \times 10^{-6**}$
	-1	1.0	**0.69**	1.7×10^{-1}
	2	0.85	**0.81**	$1.5 \times 10^{-12**}$
$N = 7$, $\sigma^2 = 1$				
	0	0.67	**0.47**	$2.1 \times 10^{-5**}$
	0.5	0.67	**0.47**	$3.0 \times 10^{-4**}$
	-1	0.65	**0.47**	$1.7 \times 10^{-2*}$
	2	0.73	**0.64**	$5.9 \times 10^{-14**}$
$N = 15$, $\sigma^2 = 1$				
	0	0.46	**0.36**	$7.4 \times 10^{-5**}$
	0.5	0.51	**0.38**	$6.6 \times 10^{-7**}$
	-1	0.49	**0.34**	$5.5 \times 10^{-5**}$
	2	0.51	**0.39**	$6.9 \times 10^{-10**}$
$N = 3$, $\sigma^2 = 5$				
	0	3.3	**0.71**	$4.5 \times 10^{-9**}$
	0.5	2.6	**0.77**	$2.2 \times 10^{-12**}$
	-1	3.0	**0.89**	$6.9 \times 10^{-9**}$
	2	3.7	**1.5**	$8.7 \times 10^{-31**}$
$N = 7$, $\sigma^2 = 5$				
	0	2.3	**0.75**	$2.1 \times 10^{-10**}$
	0.5	2.4	**0.81**	$2.7 \times 10^{-11**}$
	-1	2.2	**0.88**	$6.6 \times 10^{-7**}$
	2	2.3	**1.2**	$8.7 \times 10^{-31**}$
$N = 15$, $\sigma^2 = 5$				
	0	1.7	**0.61**	$3.5 \times 10^{-9**}$
	0.5	1.8	**0.62**	$5.2 \times 10^{-10**}$
	-1	1.9	**0.72**	$4.1 \times 10^{-7**}$
	2	1.6	**0.82**	$2.4 \times 10^{-24**}$

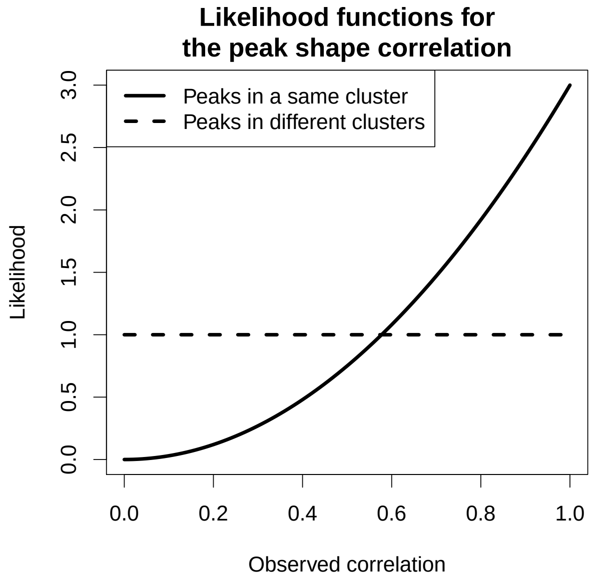

Figure 1: Probability density of the peak shape correlations. Peaks in same cluster have a high probability for high values of their mutual correlation (solid line). Peaks in different clusters are expected to have a low or entirely unobserved value of peak shape correlation (dashed line).

4 Benchmark data set with known changes of concentration

4.1 Data

- Data and the experiment are described in the publication [3]

4.1.1 Raw mass spectrometry data

- Used to extract the peak intensities and peak shape correlations
- Available at
  http://cri.fmach.eu/Research/Computational-Biology/Biostatistics-and-Data-Management/download/data/Spiked-Apple-Data. [Accessed 11.06.2013.]

4.1.2 Pre-processed and annotated intensity data

- Used to extract the peak locations, annotations and ground-truth perturbations
- Intensity data not used
- Available in the R-package `BioMark`
  - Version 0.4.1 used

- Available at
  http://cran.r-project.org/web/packages/BioMark/

4.2 Pre-processing in MZmine

4.2.1 Peak detection

- Peak list file:
  - m/z and RT values of the detected peaks from the R-package `BioMark`
  - `apple-peak_list-positive.csv`
  - `apple-peak_list-negative.csv`
- Field separator: `,`
- Ignore first line: `No`
- Intensity tolerance: `30.0` %
- m/z tolerance: `0.0010` m/z or `5.0` ppm
- Retention time tolerance: `1.0` *absolute (min)*

4.2.2 Peak alignment

- m/z tolerance: `0.0020` m/z or `10.0` ppm
- Weight for m/z: `70`
- Retention time tolerance `2.0` *absolute (min)*
- Weight for RT: `50`
- Require same charge state: `No`
- Require same ID: `Yes`
- Compare isotope pattern: `No`

4.2.3 Peak shape correlations

- Modified version of MZmine 2 used
- Time window: `0.05` (min)

4.3 Clustering

- Parameters of the beta prior distribution for the observed shape correlation values
  - Within a cluster: $a_{\text{in}} = 2$, $b_{\text{in}} = 1$
  - Between clusters: $a_{\text{out}} = 1$, $b_{\text{out}} = 1$
- Prior probability of missing value in the shape correlation data
  - Within a cluster: $p_0^{\text{in}} = 0.25$

– Between clusters: $p_0^{\text{out}} = 0.99$

- Least-squares clustering is picked from the set of Gibbs samples as proposed by [2] (Eq. 10.10) and used for the analysis that follows
- If cluster k contains annotated peaks from compound c, it is annotated as the compound c
- Clusters with annotated peaks: Table 2

Table 2: Model 1 identifies clusters that are exclusive to one annotated compound in both the positive (a) and negative (b) ion modes.

**Cluster**	**Number of peaks in the cluster**				
	Annotated peaks				Total
	cat	epicat	phlorid	querc3rham	
b) Positive ion mode					
1	2	-	0	0	27
2	0	-	8	0	9
3	0	-	1	0	1
4	0	-	0	2	6
5	0	-	0	2	16
6	0	-	0	1	1
7	0	-	0	1	1
b) Negative ion mode					
1	-	2	0	0	12
2	-	0	10	0	19
3	-	0	0	5	18

4.4 Covariate effects

4.4.1 Pre-processing

- Log-transformation of the intensity data (natural logarithm)
- Mean of the control sample group subtracted from the intensity data of each peak (control group-based centering)
- Peaks with a standard deviation of zero (constant observed value) throughout the samples removed from the data

4.4.2 Analysis

- Model 1: Posterior mean of the covariate effects computed from the Gibbs samples
- Single-peak approach:
  – Difference of means between the treatment and control group is computed for each cluster and treatment group

  - For annotated clusters, each annotated peak is used
  - For non-annotated clusters, the strongest peak by the mean of the control group (before subtracting the mean at the pre-processing) is used

- Comparison of performance between the approaches
  - Result: Table 3
  - Squared error to the ground truth change (0, 0.2, 0.4 or 1.0) is computed for each cluster/peak by both the approaches
  - Statistical significance of the difference between the mean squared errors (MSE) of the two approaches is tested by one-sided paired t-test
    * Null-hypothesis: No difference between the MSEs
    * Alternative hypothesis: The MSE of Model 1 is smaller

Table 3: Model 1 yields a more accurate quantification of the covariate effects for the spike-in compounds as well as for the unchanged non-annotated compounds in the benchmark experiment. Root mean squared error (RMSE) between the inferred and true covariate effects is smaller for Model 1 (All peaks) than for the single-peak approach (Single peak) at all magnitudes of the true effect (rows), in both the positive (a) and negative (b) ion modes. Statistical significance of the differences between errors is evaluated using the one-sided paired t-test on the null-hypothesis of equal MSEs. Significant differences at confidence levels 95 % and 99 % are highlighted by symbols "*" and "**," respectively.

**True covariate effect**	**RMSE** Single peak	**RMSE** All peaks	**Adjusted p-value of the difference**
a) Positive ion mode			
0 %	0.37	**0.27**	$< 2.2 \times 10^{-16**}$
20 %	0.38	**0.19**	$7.3 \times 10^{-4**}$
40 %	0.41	**0.27**	$1.2 \times 10^{-2*}$
100 %	0.95	**0.82**	6.6×10^{-2}
b) Negative ion mode			
0 %	0.38	**0.28**	$< 2.2 \times 10^{-16**}$
20 %	0.40	**0.18**	$9.2 \times 10^{-4**}$
40 %	0.52	**0.27**	$3.2 \times 10^{-4**}$
100 %	0.77	**0.60**	5.0×10^{-2}

5 Lipidomic data from a gene silencing study

5.1 Data

- Ultra performance liquid chromatography-mass spectrometry (UPLC)

- Ion mode: negative
- Experimental setup
  - Silenced genes:
    * ACACA (acetyl-CoA carboxylase α)
    * ELOVL1 (elongation of very long chain fatty acid-like 1)
    * FASN (fatty acid synthase)
    * INSIG1 (insulin-induced gene 1)
    * SCAP (sterol regulatory element-binding protein cleavage-activating protein)
    * SCD (stearoyl-CoA desaturase)
    * THRSP (thyroid hormone-responsive protein)
    * (Ineffective control silencing)
  - Time points:
    * 48 hours
    * 72 hours
  - Replicates in each treatment-time point category: 2
  - Total number of samples: 32
- The raw data is available at http://research.ics.aalto.fi/mi/software/peakANOVA/
- Data and the experiment described in more detail in the publication [4]

5.2 Pre-processing in MZmine 2

5.2.1 Peak detection

- Default settings

5.2.2 Peak alignment

- Default settings

5.2.3 Peak shape correlations

- Modified version of MZmine 2 used
- Time window: `0.05` (min)

5.3 Normalization

Intensities of a sample were normalized by

- intensities of standard compounds in the sample (standard compound normalization)
- amount of protein contained in the sample (tissue normalization)

5.4 Clustering

- Parameters of the beta prior distribution for the observed shape correlation values
  - Within a cluster: $a_{\text{in}} = 3$, $b_{\text{in}} = 1$
  - Between clusters: $a_{\text{out}} = 1$, $b_{\text{out}} = 1$
- Prior probability of missing value in the shape correlation data
  - Within a cluster: $p_0^{\text{in}} = 0.01$
  - Between clusters: $p_0^{\text{out}} = 0.5$
- Least-squares clustering is picked from the set of Gibbs samples as proposed by [2] (Eq. 10.10) and used for the analysis that follows

5.5 Stability of clustering

- Dirichlet process concentration parameter grid: $\alpha_{\text{DP}} = \{0.01, 0.1, 1, 10, 100\}$
- Result: Figure 2

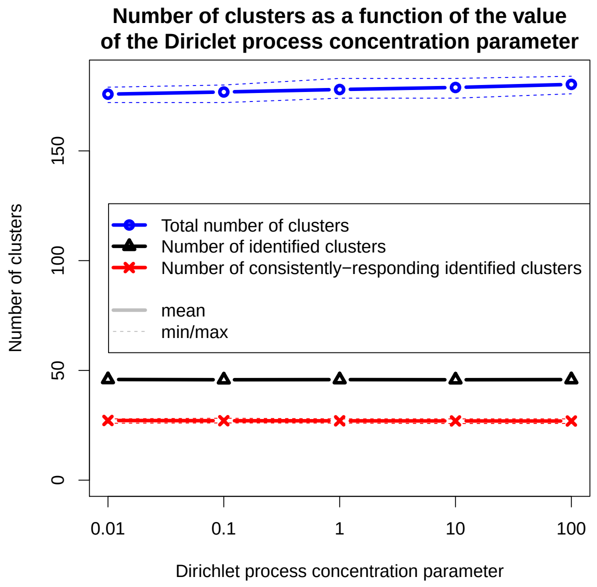

Figure 2: The clustering inferred from the lipidomic gene silencing data is insensitive to the value of the Dirichlet process concentration parameter (α_{DP}) and thus also the inferred covariate effects remain unchanged by the parameter. Total number of clusters (blue line) increases slightly as the number as the value of parameter increases on the logarithmic scale. The performance – the proportion of consistently-responding clusters (red line) among all identified clusters (red line) – remains unchanged.

5.6 Covariate effects

- Pre-processing
  - Log-transformation of the intensity data (natural logarithm)
  - Mean of the control sample group subtracted from the intensity data of each peak (control group-based centering)
  - Peaks with a standard deviation of zero (constant observed value) throughout the samples removed from the data

5.7 Consistency of effects

- Families of lipids included and the number of annotated lipids with two or more peaks identified:
  - Phosphatidylcholines (PC): 7
  - Phosphatidylethanolamines (PE): 4
  - Sphingomyelins (SM): 3
- Cross-validation
  - Number of folds: 3
  - Number of randomizations of the fold assignments and artificial noise: 100

5.8 Robustness of effects

- All identified peaks included (both annotated and non-annotated)
- Number of randomizations of artificial noise: 100

5.8.1 Added noise

- Upper bound for the signal-to-noise ratio: Table 4

References

[1] Y. Benjamini and Y. Hochberg. Controlling the false discovery rate: a practical and powerful approach to multiple testing. *J Roy Stat Soc B Met*, 57(1):289–300, 1995.

[2] D. B. Dahl. *Bayesian Inference for Gene Expression and Proteomics*, chapter Model-based clustering for expression data via a Dirichlet process mixture model, pages 201–218. Cambridge University Press, Cambridge, 2006.

[3] P. Franceschi, D. Masuero, U. Vrhovsek, F. Mattivi, and R. Wehrens. A benchmark spike-in data set for biomarker identification in metabolomics. *J Chemometr*, 26(1-2):16–24, 2012.

Table 4: Upper bound for the signal-to-noise ratio in the lipidomic experiment, where synthetic noise was added to the data with the original variance of $\sigma = 1$. In reality, the signal-to-noise ratio is lower as the original data is noisy as well.

**Added noise (σ)**	**Upper bound for the signal-to-noise ratio**
0	1.0
0.001	0.999999
0.01	0.9999
0.1	0.99
0.2	0.96
0.3	0.92
0.5	0.80
0.75	0.64
1.0	0.50
1.5	0.31
2.0	0.20
3.0	0.10
5.0	0.038
10	0.0099

[4] M. Hilvo, C. Denkert, L. Lehtinen, B. Müller, S. Brockmöller, T. Seppänen-Laakso, J. Budczies, E. Bucher, L. Yetukuri, S. Castillo, E. Berg, H. Nygren, M. Sysi-Aho, J. Griffin, O. Fiehn, S. Loibl, C. Richter-Ehrenstein, C. Radke, T. Hyötyläinen, O. Kallioniemi, K. Iljin, and M. Orešič. Novel theranostic opportunities offered by characterization of altered membrane lipid metabolism in breast cancer progression. *Cancer Res*, 71(9):3236–3245, 2011.

[5] T. Pluskal, S. Castillo, A. Villar-Briones, and M. Orešič. MZmine 2: modular framework for processing, visualizing, and analyzing mass spectrometry-based molecular profile data. *BMC Bioinformatics*, 11(1):395, 2010.

[6] T. Suvitaival, S. Rogers, and S. Kaski. Stronger findings from mass spectral data through multi-peak modeling. Submitted.
